# Mapping Conditional Associations Among Wellbeing, Distress, Spirituality, and Yoga Practice: A Network Analysis

**DOI:** 10.3390/healthcare14152293

**Published:** 2026-07-29

**Authors:** Dorottya Bosnyák, Gusztáv József Tornóczky, Attila Szabo

**Affiliations:** 1 Doctoral School of Education, Faculty of Education and Psychology, ELTE Eötvös Loránd University, 1053 Budapest, Hungary; dorottyabos@student.elte.hu; 2 Institute of Health Promotion and Sport Sciences, Faculty of Education and Psychology, ELTE Eötvös Loránd University, 1117 Budapest, Hungary; tornoczky.gusztav@ppk.elte.hu; 3 Physical Education and Exercise Centre, Medical School, University of Pécs, 7624 Pécs, Hungary; 4 Department of Psychology and Health Management, Faculty of Health and Sport Sciences, Széchenyi István University, 9026 Győr, Hungary

**Keywords:** mind–body exercise, wellbeing, spirituality, perceived health, distress, yoga

## Abstract

**Highlights:**

**What are the main findings?**

- Network analysis revealed distinct but interconnected wellbeing, distress, and spirituality domains among yoga practitioners.
- Perceived health and yoga practice frequency were uniquely associated with wellbeing beyond psychological distress. Environmental spirituality was a significant correlate in the main model, although supplementary analyses indicated that this association depended on model specification.

**What are the implications of the main findings?**

- The findings contribute to a multidimensional view of wellbeing that integrates psychological, health-related, and spiritual factors.
- Network approaches may help identify potential correlates for yoga-based health promotion and wellbeing interventions.

**Abstract:**

**Background/Objectives**: Yoga practice is associated with enhanced wellbeing and reduced psychological distress; however, the conditional interrelations among yoga frequency, perceived health, wellbeing, distress, and spirituality remain insufficiently understood. This study aimed to map these associations and identify variables uniquely relevant to wellbeing among yoga practitioners. **Methods**: A cross-sectional online survey was conducted among 326 yoga practitioners (94.2% women; Mage = 39.84, SD = 14.81). A Gaussian graphical model with EBIC-graphical LASSO regularization was used to estimate conditional associations among eight variables. Hierarchical multiple regression was conducted to identify independent predictors of wellbeing. **Results**: The network retained 20 of 28 possible edges (sparsity = 0.286) and revealed three clusters: health wellbeing (yoga frequency, perceived health, and wellbeing), distress (anxiety and stress), and spirituality. Global experiential spirituality showed the highest strength and expected influence, whereas WHO-5 wellbeing was the most central node in terms of betweenness and closeness. Stability was acceptable to excellent (CS coefficients: strength = 0.70, closeness = 0.60, betweenness = 0.40). The association between yoga frequency and wellbeing persisted independently of distress. The regression model explained 48.1% of the variance in wellbeing (adjusted R^2^ = 0.469, *p* < 0.001). Higher environmental, but not transcendent, spiritual health (β = 0.136), better perceived health (β = 0.256), and more frequent yoga practice (β = 0.104) uniquely predicted higher wellbeing, whereas depression (β = −0.362) and stress (β = −0.198) uniquely predicted lower wellbeing. The environmental spirituality association was significant in this main model but, in a supplementary model that included all four experiential spirituality domains, was attenuated and depended on model specification. **Conclusions**: Wellbeing among yoga practitioners is a multidimensional construct uniquely associated with lower levels of depression and distress, as well as self-rated health and environmental spirituality. Depression and stress showed negative associations with wellbeing, whereas perceived health, regular yoga practice, and environmental spirituality showed positive associations. These findings highlight potential correlates of subjective wellbeing among yoga practitioners and underscore the importance of considering both distress-related and positive health-related factors, including perceived health, regular yoga practice, and environmental spirituality (interpreted with caution given its sensitivity to model specification), in future research.

## 1. Introduction

Throughout its vast history, yoga has taken various forms, including philosophies, interpretations, and practices [1]. However, at its origin, the central concept of yoga highlights the “nature of the mind and its afflictions (Kleshas)” [2]. Yoga’s primary goal is to gain control over the mind, allowing one to control all perceptions and, therefore, be bound by nothing, reaching ultimate freedom [3,4]. Such freedom is what would be classified as wellbeing in contemporary terminology.

More specifically, as defined by the World Health Organization (WHO), wellbeing “encompasses quality of life and the ability of people and societies to contribute to the world with a sense of meaning and purpose” [5]. Yoga, as a mind–body intervention, incorporates meaning-making and purpose-making, as well as physical wellbeing, into its practices. The connection between yoga and wellbeing—while rooted in ancient philosophies—is also backed by contemporary science. With the rise in yoga’s popularity, research on yoga has grown as well [6]. The accumulating body of research increasingly identifies yoga as a lifestyle and practice with multifaceted positive effects, specifically on overall wellbeing [7,8]. This becomes especially relevant given that, in 2025, the WHO estimated that about 5.7% of the world’s adult population suffered from depression [9,10], and approximately 4.4% suffered from anxiety [9,11]. When such numbers are combined with the low prevalence and availability of institutionalized treatment, alternative remedies are crucial, and yoga is among the most used [12,13,14]. These observations are best understood through a conceptual framework that distinguishes between distress-related and health-promoting dimensions of mental health.

This study integrates two complementary theoretical perspectives that provide the conceptual lens for examining yoga-related wellbeing. First, Keyes’ [15] Complete State Model of Mental Health posits that wellbeing and psychological distress are related but distinct dimensions. Specifically, the absence of life stress does not automatically translate into high wellbeing, and positive factors must be actively present for flourishing to occur. This model provides the rationale for examining both distress-related (depression, anxiety, and stress) and health-promoting variables (yoga practice, perceived health and spirituality) as simultaneous but independently contributing predictors of wellbeing. Second, building on Coan’s [16] distinction among five discrete dimensions of wellbeing, the study treats spirituality not as a byproduct of practice but as a potentially distinct pathway through which yoga may support wellbeing. Together, these two theoretical frameworks predict that wellbeing among yoga practitioners is a multidetermined outcome uniquely associated with the interplay between distress reduction and the presence of positive experiential factors, forming a system of conditional associations that network analysis is uniquely suited to capture [17].

Within the distress dimension of this model, the use of yoga for distress reduction is not unjustified, as its positive effects have been documented in numerous research studies and clinical trials. Overall, yoga has been linked to improvements in physical and mental health outcomes, as well as overall quality of life [12]. Yoga has also been empirically linked to reductions in depression and anxiety symptoms across diverse age groups [18,19,20]. Yoga’s positive stress-reducing effects have also been corroborated by psychobiological measurements, including reductions in blood pressure, heart rate, cortisol, and cytokine levels, which are linked to improved overall wellbeing [21].

On the health-promoting dimension of the model, wellbeing is much more complex than just its psychological aspect. Since the 1970s, efforts have been made to establish wellbeing as a multidimensional construct [16,22]. Recently, contemporary research [23,24,25] has established spirituality as a core construct of wellbeing, as Coan [16] had already theorized. Spiritual wellbeing is achieved when oneness with the environment is attained, accompanied by sufficient self-awareness and introspection. Those with high spirituality live in harmony with their surroundings, are at peace with God and enjoy life [26,27]. Yoga, as an inherently spiritual, transcendent practice, aims to cultivate spiritual awareness [28]. Intensive practice has been linked to improvements in various aspects of spirituality, such as compassion and spiritual orientation, as well as improved mood [29]. Even in Western societies, which often emphasize the physical fitness aspect of yoga, regular practice has been associated with increased spiritual interest, spiritual wellbeing, as well as overall health [30]. Subjective wellbeing (SWB) encompasses an individual’s cognitive, behavioral, and affective self-ratings. Yoga has been linked to subjective wellbeing, often measured through perceived health and wellbeing [31,32]. Research suggests that whilst longer yoga practice has been associated with greater improvements in self-rated wellbeing [31], even relatively short yoga experiences can have a measurable positive effect on perceived health [32]. Yoga practice has even been associated with more positive illness perceptions among chronically ill participants, alongside improved mental wellbeing and quality of life [33].

Whilst the relationship between yoga and wellbeing has been investigated from various angles, the complex, conditional interrelations among yoga practice, perceived health, wellbeing, distress, and especially spirituality remain underinvestigated and therefore less understood. Systematic reviews have noted that research on yoga rarely incorporates spiritual variables, with most studies focusing on psychophysiological and therapeutic benefits while neglecting the spiritual dimension [29,30]. Moreover, available studies tend to examine these constructs in isolation or in pairs rather than as an interconnected system. For instance, Bös et al. [34] found that yoga practice was associated with increased spiritual wellbeing but not with psychological wellbeing or psychological distress. Moreover, a study by Gaiswinkler and Unterrainer [35] found that yoga has the greatest effects on psychological and spiritual wellbeing when it forms part of the practitioner’s worldview. Such findings, amongst others [36,37], suggest that the interrelations among yoga, spirituality, wellbeing, and psychological distress are more complex than simple positive associations. Therefore, this study aims to map these associations and identify variables uniquely relevant to wellbeing among yoga practitioners.

Accordingly, we hypothesized that yoga practice frequency, perceived health, and spirituality would show positive unique associations with wellbeing beyond and independent of the negative contributions of depression, anxiety, and stress, reflecting the dual spectra principle that wellbeing is sustained by both the reduction in distress and the activation of health-promoting and spiritual resources. In network terms, we expected wellbeing to show direct associations with both distress-related nodes (depression, anxiety, and stress) and health-promoting nodes (yoga practice, perceived health, and spirituality).

The present manuscript extends previous research by applying network analysis to map the interconnected structure among yoga practice, perceived health, wellbeing, distress, and spirituality. Prior work has examined these variables in pairs or through regression analyses; comparatively, the present methodology offers two advantages. Firstly, it shows the conditional dependence structure of the variables, separating direct relationships after accounting for shared variance across the system. Secondly, it identifies system-level properties that cannot be captured by variable-focused approaches. Through the combination of network analysis and hierarchical regression, we hope to address both the system structure and the unique explanatory relevance that each variable has for wellbeing. This approach advances theoretical understanding by testing Keyes’ Complete State Model as a system of interacting health-promoting, spiritual, and distress-related factors, rather than as a set of separate associations.

## 2. Materials and Methods

### 2.1. Study Design and Sample Size Calculation

The present study employed a cross-sectional, online survey design targeting Hungarian adults. Data were collected between October 2022 and April 2025 using a self-administered electronic questionnaire administered via the Qualtrics research platform [38]. Consistent recruitment procedures, eligibility criteria, and study protocols were maintained throughout the entire data collection period. Recruitment proceeded in intermittent waves across this period, with approximately 11% of the sample (36 participants) recruited in early 2025. The analytic dataset was subsequently finalized and locked prior to statistical analysis. The study was conducted under ethics approval No. 2022/145-2; this approval specified an expected completion date of December 2024 but contained no fixed expiry date and remained in force throughout the data collection period, including for participants recruited in early 2025. Preliminary inspection revealed no evidence of systematic changes in the principal study variables across the recruitment period: collection date showed only small associations with WHO-5 wellbeing, global ideal spirituality, and weekly yoga frequency (all |r| ≤ 0.13), none of which remained significant after correction for multiple comparisons.

An a priori sample size calculation was conducted with G*Power software version 3.1.9.7 [39] for multiple linear regression using seven predictors, with α = 0.05 and statistical power (1 − β) set at 0.80. To detect a medium effect size according to Cohen’s convention (f^2^ = 0.15), a minimum sample size of N = 103 was required. To detect a smaller effect (f^2^ = 0.05), a minimum sample size of N = 295 was required. The final sample of N = 326 therefore exceeded the required sample size for detecting small-to-moderate effects in the planned regression model. No universally applicable a priori GGM calculation was used to calculate the minimum sample size for the network analysis; adequacy was evaluated empirically through edge-accuracy and case-dropping stability analyses.

### 2.2. Participants

#### 2.2.1. Sample Characteristics

Participants were recruited using a combination of convenience and snowball sampling strategies. Initially, psychology students at Eötvös Loránd University invited individuals from their personal networks to participate in a field practice course. Participants were subsequently encouraged to share the survey within their own social networks. In addition, the Hungarian Yoga Teachers’ Association supported recruitment by distributing the study invitation among its members and encouraging further participation among yoga practitioners. Although these recruitment strategies facilitated access to a geographically diverse sample of yoga practitioners, they may also have introduced self-selection bias and limited the representativeness of the study population. These issues are further considered in the Limitations section.

Eligibility criteria required participants to be aged 18 years or older, to self-identify as healthy, and to be currently practicing yoga (self-reported). All participants provided informed consent prior to participation. Responses were excluded if the questionnaire was incomplete or if the eligibility criteria were not met (e.g., underage respondents or non-yoga practitioners). Participation was voluntary and anonymous, and no financial or other incentives were provided.

A total of 326 participants were included in the analysis, with a mean age of 39.84 years (SD = 14.81; range: 18–82). The sample characteristics are presented in Table 1. The sample was predominantly female (94.2%). Participants were generally highly educated, with nearly two-thirds holding a tertiary degree (63.8%) and approximately one-third having completed secondary education. In terms of family status, the largest proportion of respondents were married (41.1%), followed by single individuals (24.8%) and those in a relationship or civil partnership (24.5%).

Regarding place of residence, the largest proportion of participants lived in the capital city (38.0%), although respondents were represented across all settlement types. Importantly, participants were recruited from all counties of Hungary, indicating nationwide coverage. While a small proportion of respondents (4.3%) resided abroad, all were Hungarian nationals. With respect to employment status, nearly half of the sample was employed (46.3%), with additional representation from entrepreneurs (22.7%) and students (19.9%). Overall, the sample can be characterized as predominantly female, highly educated, and geographically diverse.

#### 2.2.2. Yoga-Related Characteristics of the Sample

Yoga-related characteristics of the participants are presented in Table 1 and Table S1. Most respondents were yoga practitioners (68.7%), while nearly one-third were certified yoga instructors, and most participants reported engaging in additional physical activity alongside yoga (82.5%) (Table 1). Regarding practice frequency, yoga components were most commonly performed once per week across all domains. This pattern was particularly pronounced for meditation, with more than half of participants reporting weekly practice (54.9%), compared with lower proportions for asana (31.6%), pranayama (38.7%), and relaxation (37.1%) (Table S1).

Moderate engagement (i.e., two to three sessions per week) was also common across components, whereas higher-frequency practice (four or more times per week) was less prevalent. Daily practice was reported by a minority of participants, ranging from 9.8% for meditation to 12.3% for relaxation. Overall, these findings indicate that while participants regularly engaged in multiple components of yoga practice, participation tends to be moderate, with weekly and mid-range patterns predominating across domains. In the present study, yoga practice was operationalized primarily through weekly practice frequency, as this represented the most consistently reported indicator of yoga engagement across participants. Other characteristics of practice, including duration, years of experience, style, intensity, and practice setting, were not incorporated into the present analyses and warrant investigation in future studies.

### 2.3. Instruments

In the first section of the questionnaire, participants provided sociodemographic information, including age, gender, educational attainment, marital status, and place of residence. Participants also reported yoga-related characteristics, including their role (yoga practitioner or instructor), engagement in additional physical activity, and the frequency of practicing different components of yoga (asana, pranayama, relaxation, and meditation).

#### 2.3.1. Depression, Anxiety, and Stress

Psychological distress was assessed using the 21-item version of the Depression Anxiety Stress Scales (DASS-21) [40]. The instrument comprises three subscales measuring depression, anxiety, and stress, each consisting of seven items. Participants rated the extent to which they experienced each symptom during the past week on a 4-point Likert scale ranging from 0 (Did not apply to me at all) to 3 (Applied to me very much or most of the time), with scores representing symptom severity rather than clinical diagnosis. The scale includes items such as “I tended to over-react to situations” and “I found it difficult to relax.” The DASS-21 has demonstrated good validity and reliability across clinical and non-clinical samples [41,42,43]. In the present study, internal consistency was high for all subscales (Depression α = 0.89, ω = 0.89; Anxiety α = 0.85, ω = 0.86; Stress α = 0.87, ω = 0.87).

#### 2.3.2. Self-Rated Health

Perceived health status was assessed using a single-item measure of overall health. Participants rated their health on a 5-point Likert scale ranging from very poor (1) to excellent (5). The measure included the question, “How would you rate your overall health?” This measure has been widely used in Hungarian population-based research [44]. Single-item self-rated health measures are also extensively employed in epidemiological and public health research because they provide a valid global indicator of perceived health and have consistently demonstrated predictive validity for a wide range of health outcomes.

#### 2.3.3. Spiritual Wellbeing

Spiritual wellbeing was assessed using the Spiritual Health and Life-Orientation Measure (SHALOM) [26]. The scale consists of 20 items across four domains: personal, communal, environmental, and transcendental spirituality. Each item is rated twice to capture both ideal and experiential levels of spiritual wellbeing. Responses are provided on a 5-point Likert scale ranging from 1 (Very low) to 5 (Very high). The scale includes items such as “Inner peace,” “Kindness towards other people,” “Connection with nature,” and “Peace with God,” representing the four domains assessed by the instrument. In the present study, overall scale scores (general factor) were calculated separately for the two evaluation levels (ideal and experiential spirituality), and regression analyses were conducted using both the total scale scores for ideal and experiential spirituality and the four domain-specific subscales. The Hungarian version has demonstrated good psychometric properties, supporting a bifactor structure [45]. In the present study, internal consistency coefficients indicated excellent reliability (Ideal α = 0.93, ω = 0.92; Experiential α = 0.93, ω = 0.92; Ideal domains α = 0.84–0.97, ω = 0.84–0.97; Experiential domains α = 0.85–0.97, ω = 0.85–0.97).

#### 2.3.4. Subjective Wellbeing

Subjective wellbeing was measured using the validated Hungarian version of the WHO Well-Being Index (WHO-5) [46], a brief 5-item self-report measure assessing positive emotional wellbeing over the past two weeks. Responses are rated on a 4-point Likert scale ranging from 0 (Not at all true) to 3 (Completely true). Summed total scores were calculated and used in all statistical analyses, with higher scores indicating greater subjective wellbeing. Example items include “I have felt calm and relaxed” and “I have felt active and vigorous.” The Hungarian version has demonstrated good reliability and validity [47]. In the present study, internal consistency was good (α = 0.78, ω = 0.77).

Although the original WHO-5 is often scored on a 0–5 scale and transformed to a 0–100 range, the Hungarian version validated by Susánszky et al. [47] employs the 0–3 response format. Based on this validation, we consider the present scoring to be appropriate and interpretable, and we presume comparability with the broader WHO-5 literature within the limits of the different response scaling.

### 2.4. Statistical Analysis

All statistical analyses were conducted using IBM SPSS Statistics (version 30) and JASP (version 0.95). Descriptive statistics were used to summarize sociodemographic and yoga-related variables. Continuous variables are reported as means and standard deviations, while categorical variables are presented as frequencies and percentages. The sample was treated as a single, homogeneous group for all analyses, and no subgroup comparisons within yoga practitioners were conducted.

To examine the multivariate relationships among psychological, health, and yoga-related variables, a Gaussian graphical model (GGM) was estimated using regularized partial correlations, an approach grounded in network theory that conceptualizes psychological constructs as systems of interacting components [17,48]. The eight variables included in the network were selected based on theoretical relevance, guided by Keyes’ Complete State Model of Mental Health [15] and the multidimensional conceptualization of wellbeing [16], to represent the core constructs of interest: yoga engagement, perceived health, subjective wellbeing, psychological distress, and spirituality. Network estimation was conducted using the graphical least absolute shrinkage and selection operator (graphical LASSO) with extended Bayesian information criterion (EBIC) model selection [49], yielding sparse and interpretable network structures.

Network characteristics were examined using centrality and clustering indices. Centrality measures included strength, expected influence, closeness, and betweenness. Clustering coefficients were calculated using multiple algorithms (Barrat, Onnela, Watts–Strogatz, and Zhang methods) to assess local interconnectedness. Node distinguishability was evaluated using pairwise centrality difference tests.

To assess network robustness, bootstrap procedures were applied in accordance with established guidelines for psychological network analysis [50]. Edge-weight accuracy was evaluated using nonparametric bootstrap resampling, while centrality stability was assessed using case-dropping subset bootstrap methods. Correlation stability (CS) coefficients were computed to quantify the stability of centrality estimates; values above 0.25 indicate acceptable stability, and values above 0.50 indicate good stability.

Hierarchical multiple regression analysis was conducted to examine the predictive roles of spiritual health, psychological distress (depression, anxiety, and stress), weekly yoga frequency, and perceived health in explaining subjective wellbeing (WHO-5). Predictors were entered in blocks based on theoretical considerations. Model fit was evaluated using R^2^ and adjusted R^2^ values, and changes in explained variance (ΔR^2^) were assessed at each step. Multicollinearity was assessed using variance inflation factors (VIFs) and tolerance values, whereas autocorrelation of residuals was evaluated using the Durbin–Watson statistic. In addition, a supplementary correlation matrix of all predictors is provided to facilitate the interpretation of the regression coefficients. All analyses were conducted in accordance with current best practices in psychological network analysis. Statistical significance was set at *p* < 0.05 (two-tailed).

Different operationalizations of spirituality were used in the two analytical approaches because they addressed different research questions. To maintain a parsimonious and interpretable network structure, the network analysis included the global ideal and experiential spirituality scores as indicators of the broader spirituality constructs. In contrast, the hierarchical regression analysis examined individual spirituality dimensions to identify those showing unique associations with subjective wellbeing. This complementary analytical strategy allowed both an overall examination of the conditional association structure and a more detailed evaluation of the independent relationships between specific spirituality dimensions and wellbeing. Within the regression, the two experiential domains entered as spirituality predictors in the main model—experiential environmental and experiential transcendent spirituality—were examined to contrast a nature-connected, lived dimension of spirituality with a transcendent, belief-based dimension, a distinction developed in the Discussion. All four experiential domains showed significant positive bivariate associations with wellbeing (see Table S8); because the two domains entered in the main model constitute a theoretically motivated subset rather than a fully pre-specified single model. The domain-level regression is reported as exploratory. To assess the robustness of the environmental spirituality finding, a sensitivity analysis including all four experiential domains and, separately, the global experiential spirituality score is provided in the Supplementary Material (Table S8).

### 2.5. Ethical Compliance

Ethical approval for the study was obtained from the Research Ethics Committee of the Faculty of Pedagogy and Psychology at Eötvös Loránd University (approval number: 2022/145-2). All procedures were conducted in accordance with the Declaration of Helsinki. Participants were informed about the study’s aims and procedures prior to participation and provided electronic informed consent before accessing the questionnaire. Participation was voluntary and anonymous, and respondents had the right to withdraw at any time without consequence. All data were collected in a de-identified manner and used exclusively for research purposes to ensure confidentiality and data protection.

## 3. Results

### 3.1. Network Structure

**Figure 1:**
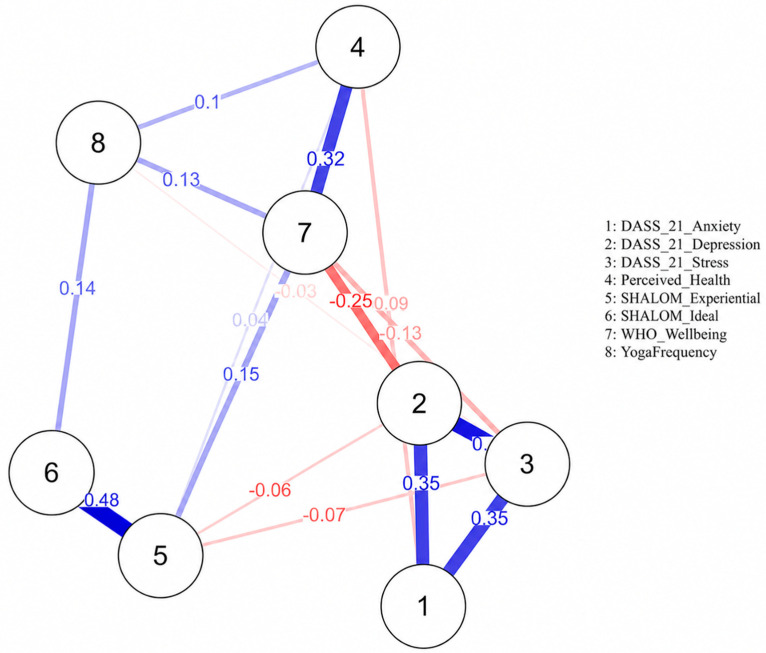
Estimated Psychological Network of Yoga, Health, Wellbeing, Distress, and Spirituality Variables. Note. Edge thickness is proportional to the regularized partial correlation weight. Blue edges indicate positive conditional associations; red edges indicate negative conditional associations. Maximum edge weight = 0.69. Node labels: 1 = DASS-21 Anxiety; 2 = DASS-21 Depression; 3 = DASS-21 Stress; 4 = Perceived Health; 5 = SHALOM Experiential; 6 = SHALOM Ideal; 7 = WHO-5 Wellbeing; 8 = Yoga Frequency.

A Gaussian graphical model was estimated for eight nodes: yoga frequency, perceived health, WHO-5 wellbeing, DASS-21 depression, DASS-21 anxiety, DASS-21 stress, spiritual ideal, and spiritual experiential. The regularized partial correlation network retained 20 of 28 possible edges (sparsity = 0.286), indicating a moderately dense structure (see Table S2 and Figure 1).

Three interpretable subnetworks were apparent within the network, although these were not fully distinct and showed several cross-domain connections. First, a health and wellbeing cluster included positive connections among yoga frequency, perceived health, and WHO-5 wellbeing, with perceived health and WHO-5 wellbeing showing the largest positive edge weight in this cluster (0.412), followed by yoga frequency–WHO-5 wellbeing (0.349) and yoga frequency–perceived health (weight = 0.348). Second, a distress cluster focused on DASS-21 anxiety and DASS-21 stress, which shared the strongest positive association in the entire network (weight = 0.686). DASS-21 depression showed conditional associations with both the distress-related variables and the spirituality variables, including positive associations with anxiety (weight = 0.105) and spiritual ideal (weight = 0.288), together with a small negative association with stress (weight = −0.041). Third, a spiritual cluster connected spiritual ideal and spiritual experiential through a positive link (weight = 0.122), while spiritual ideal showed negative conditional associations with perceived health (weight = −0.222) and WHO-5 wellbeing (weight = −0.142), and a positive conditional association with DASS-21 anxiety (weight = 0.237). The full edge weight matrix is presented in Table S3.

### 3.2. Centrality and Clustering

Standardized centrality indices are presented in Table 2. WHO-5 Wellbeing emerged as the most central node in terms of betweenness and closeness, whereas SHALOM Experiential showed the highest strength and expected influence. DASS-21 Depression showed the second-highest betweenness (z = 1.142) and closeness (z = 0.752), consistent with its location between the distress and spiritual clusters in the network plot. In terms of node strength, defined as the sum of absolute edge weights, Spirituality (Experiential) ranked highest (z = 0.983), followed closely by WHO-5 Wellbeing (z = 0.884) and DASS-21 Depression (z = 0.798). Yoga Frequency was the most peripheral node across all indices, registering the lowest standardized closeness (z = −1.444), strength (z = −1.868), and expected influence (z = −0.849). Regarding expected influence, which accounts for edge sign, Spirituality (Experiential) showed by far the largest positive value (z = 1.805), while WHO-5 Wellbeing showed the most strongly negative value (z = −1.395), indicating that these two nodes differed in the overall signed pattern of their conditional associations with adjacent nodes.

All values are standardized z-scores computed from the estimated network. Betweenness = proportion of shortest paths in the network that pass through a node; Closeness = inverse of the mean shortest path length to all other nodes; Strength = sum of absolute edge weights; Expected Influence = sum of signed edge weights (sensitive to edge direction).

Standardized clustering coefficients are presented in Table S4. Clustering reflects the degree to which a node’s neighbors are themselves interconnected. Spirituality (Ideal) showed the highest Barrat (z = 1.566) and WS (z = 0.972) clustering values, as did Yoga Frequency on WS (z = 0.972), indicating that these nodes’ neighbors tend to be mutually connected. Clustering estimates varied across algorithms. DASS-21 Anxiety showed the highest Onnela and Zhang coefficients but the lowest Barrat and WS coefficients, indicating that its local neighborhood was highly sensitive to the choice of clustering estimator.

All values are standardized z-scores. Four clustering estimators are reported: Barthélemy et al. [51], Onnela et al. [52], Watts & Strogatz [53], and Zhang & Horvath [54]. Discrepancies across estimators reflect differences in how each algorithm weights edge magnitudes.

### 3.3. Node Distinguishability

**Figure 2:**
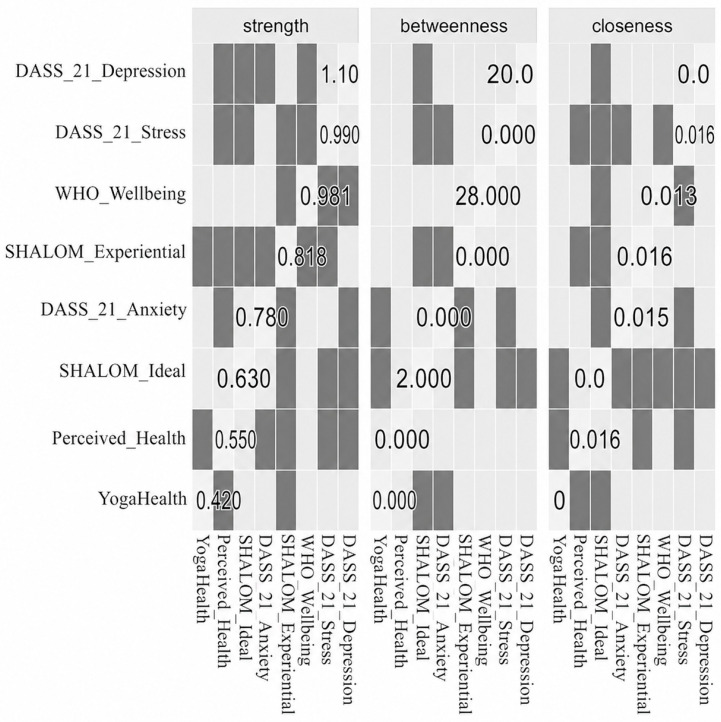
Centrality Difference Test Heatmap for Strength, Betweenness, and Closeness. Note. Each cell shows a pairwise test statistic for the difference between two nodes on the corresponding centrality index. Darker shading indicates larger test statistics (i.e., nodes that are more clearly distinguishable). White cells indicate pairs of nodes that cannot be statistically distinguished. Node order on both axes remains the same within each panel.

Figure 2 shows a heatmap of a centrality difference test comparing pairs of nodes’ strengths, betweenness, and closeness. Each cell features a test statistic that reveals whether the two nodes differ significantly on that centrality measure. Darker cells indicate pairs of nodes that are statistically distinct. For strength, SHALOM Experiential and Yoga Frequency were most clearly distinguishable from the remaining nodes, as indicated by relatively large test statistics in their respective rows and columns. On betweenness, WHO-5 Wellbeing and DASS-21 Depression showed the clearest separation from the other nodes. On closeness, WHO-5 Wellbeing was the most robustly distinguishable node. Nodes with similar centrality scores, especially those with z-scores near the network average, had smaller or near-zero test statistics, suggesting they could not be reliably distinguished from one another in this sample.

### 3.4. Network Stability

To evaluate network accuracy and stability, two bootstrap procedures were performed (Table S5). Edge-weight accuracy was assessed using 500 nonparametric bootstrap samples based on case resampling with replacement. Centrality stability was assessed using 500 case-dropping subset bootstrap samples, from which correlation-stability (CS) coefficients were derived. The CS coefficient reflects the maximum proportion of cases that can be removed while retaining, with 95% probability, a correlation of at least 0.70 between centrality estimates from the original sample and those from the subset samples [50]. Values above 0.25 indicate minimum acceptable stability, whereas values above 0.50 indicate preferable stability.

Edge stability results are presented in Figure S1. Bootstrapped confidence intervals were narrow for the strongest edges, including yoga frequency–perceived health, yoga frequency–WHO-5 wellbeing, and DASS-21 anxiety–stress, indicating that these associations were estimated with high precision. Weaker edges showed wider confidence intervals and greater overlap with zero, suggesting that smaller partial correlations should be interpreted with more caution. The ordering of edges from strongest to weakest was broadly preserved across bootstrap samples, supporting the relative ranking of edge weights.

CS coefficients for the three centrality indices are presented in Table S6. Strength obtained a CS coefficient of 0.70, and closeness obtained a CS coefficient of 0.60, both exceeding the preferred ≥0.50 threshold. Betweenness obtained a CS coefficient of 0.40, meeting the minimum acceptable threshold of ≥0.25 but falling below the preferred threshold. These results suggest that strength and closeness centrality rankings were comparatively robust to case removal, whereas betweenness was less stable and should be interpreted with greater caution, especially when comparing nodes with similar betweenness values. Overall, the stability analyses support the substantive centrality conclusions, particularly those concerning WHO-5 Wellbeing and Yoga Frequency, whose extreme centrality values rendered them readily distinguishable from other nodes across bootstrap subsets.

The CS coefficient is the maximum proportion of cases that can be removed while, with 95% probability, retaining a correlation of at least 0.70 between centrality estimates from the original sample and those from subset samples [50].

### 3.5. Hierarchical Regression

A hierarchical multiple regression (Table S7) was conducted to examine whether spiritual health, psychological distress, weekly yoga frequency, and perceived health predicted WHO-5 wellbeing among yoga practitioners (N = 326). Spiritual health variables entered at Steps 1–2 accounted for 16.9% of the variance in wellbeing, with environmental spiritual health emerging as a positive predictor, whereas transcendent spiritual health made only a small contribution. Adding depression, anxiety, and stress at Step 3 significantly improved the model and increased the explained variance to 40.7%. Weekly yoga frequency added a further 1.7% at Step 4, and perceived health added an additional 5.6% at Step 5. The final model was significant, F(7, 318) = 42.04, *p* < 0.001, and explained 48.1% of the variance in wellbeing (adjusted R^2^ = 0.469). In the final model, higher environmental spiritual health, better perceived health, and more frequent weekly yoga practice were uniquely associated with higher wellbeing, whereas higher depression and stress were uniquely associated with lower wellbeing. Transcendent spiritual health and anxiety showed no unique associations with wellbeing in the final model (Table 3). Collinearity statistics were acceptable (VIFs = 1.17–2.74), and the Durbin–Watson statistic (2.10) indicated no notable autocorrelation. Tolerance values ranged from 0.36 to 0.85, further indicating that multicollinearity was not of sufficient magnitude to compromise the stability or interpretation of the regression coefficients.

## 4. Discussion

The present study combined Gaussian graphical modeling and hierarchical regression because these methods address complementary methodological questions. Whereas the network analysis characterizes the conditional association structure among the studied variables as an interconnected system, the hierarchical regression identifies which theoretically selected variables retain unique associations with subjective wellbeing after accounting for shared variance. Together, these approaches provide a more comprehensive understanding of wellbeing among yoga practitioners than either method alone.

Network analysis offers a distinct advantage over variable-centered approaches by revealing the unique pairwise relationships among constructs while controlling for all other nodes, thereby capturing the complexity of psychological systems that traditional latent-variable models may obscure [17,48]. The estimated network was moderately dense (20 of 28 possible edges retained; sparsity = 0.286) and organized into three substantively interpretable domains, broadly consistent with the prior literature suggesting that health, distress, and spiritual constructs are related yet separable aspects of functioning [36,37,55,56]. Importantly, these conditional associations should not be interpreted as direct or causal effects but rather as unique relationships between variables after controlling for all other nodes in the network. In this sense, edges represent partial associations that may differ from bivariate relationships, particularly in the presence of conceptually overlapping constructs such as depression, anxiety, and stress. Accordingly, caution is warranted when interpreting node importance, as psychological network models capture conditional dependence structures rather than causal pathways, and centrality estimates may be influenced by model specification, sampling variability, and construct overlap [50,57,58].

The most theoretically consequential cluster for the study’s aims comprised yoga frequency, perceived health, and WHO-5 wellbeing. The positive edges linking these three nodes—with partial correlations ranging from 0.348 to 0.412—are consistent with a substantial body of evidence indicating that regular yoga practice is associated with improved self-rated health and hedonic wellbeing [13,21,31,56,59].

Importantly, these associations persisted after adjusting for the other variables included in the network, suggesting that the yoga–wellbeing connection was not fully explained by distress-related variance. The centrality analyses further showed high values for the WHO-5 wellbeing on betweenness and closeness. Moreover, SHALOM Experiential showed the highest strength and expected influence. This pattern is consistent with the WHO-5’s established sensitivity to broad aspects of psychosocial wellbeing and its cross-domain validity [46,60,61].

The peripheral positioning of yoga frequency—reflected in the lowest strength (z = −1.868) and closeness (z = −1.444) values—indicates that yoga practice is conditionally associated with relatively few other nodes after accounting for shared variance, a pattern suggesting its effects may be indirect, working primarily through perceived health and wellbeing rather than directly impinging on distress or spirituality. More generally, these and the subsequent network-based interpretations should be regarded as descriptive hypotheses derived from the observed conditional dependence structure rather than as evidence of directional or causal relationships.

The distress cluster was dominated by the strong association between DASS-21 anxiety and DASS-21 stress (weight = 0.686), replicating the well-established co-occurrence of these two symptom dimensions at the item and subscale levels [40,41,62,63]. Within the estimated network, depression was conditionally connected to both distress-related and spirituality-related variables, displaying positive associations with anxiety (weight = 0.105) and spiritual ideal (weight = 0.288). Although this pattern may be consistent with a bridging role, this interpretation should be regarded as descriptive and hypothesis-generating rather than as evidence of an underlying psychological mechanism. Taken together, these findings may indicate that depression occupies a position linking distress-related and spirituality-related variables within the estimated network, a pattern that is broadly consistent with theoretical accounts of spiritual struggle and depression [55,64]. The two spirituality nodes were connected to each other (weight = 0.122) and showed consistent negative conditional associations with perceived health and WHO-5 wellbeing, while spiritual ideal was positively associated with anxiety and depression. One possible interpretation is that stronger endorsement of ideal spiritual standards may coexist with greater psychological strain in some individuals. Several theoretical perspectives may help contextualize the pattern of associations observed in the present study. Bonelli et al. [65] suggested that high religious or spiritual standards may, under certain circumstances, be accompanied by spirituality-related guilt. Similarly, Fox and Picciotto [66] reported that psychological avoidance and spiritualizing mediated the relationship between spirituality and symptoms of depression and anxiety. Other authors have also discussed how certain forms of spirituality may become psychologically challenging when they involve rigid expectations or unresolved spiritual struggles [67,68]. Furthermore, recent research suggests that discrepancies between ideal and experiential spirituality may be associated with internal tension and reduced wellbeing, particularly when individuals experience difficulties integrating their spiritual ideals into everyday life [69]. Although these perspectives offer plausible theoretical explanations for the observed pattern of conditional associations, the present study did not directly assess these psychological mechanisms. Therefore, our findings should not be interpreted as evidence for these processes but rather as indicating that different dimensions of spirituality may relate to wellbeing and psychological distress in more complex ways than uniformly protective associations would suggest. Future studies incorporating direct measures of spiritual struggle, spiritual coping, and related psychological processes are needed to examine these potential explanatory pathways.

The stability analyses provided satisfactory support for the centrality conclusions. CS coefficients of 0.70 for strength and 0.60 for closeness exceeded the preferred threshold of 0.50, and the betweenness CS coefficient of 0.40 met the minimum acceptable criterion of 0.25. Strength and closeness therefore showed good stability, while betweenness met the minimum threshold but remained below the preferred value [50]. The edge-weight bootstrap further confirmed precision for the strongest associations in the network. Several limitations warrant acknowledgment. First, the cross-sectional design of the study precludes causal inference; the network structure captures conditional associations rather than directed influence, and longitudinal or experimental designs would be required to test whether changes in yoga frequency produce downstream changes in perceived health or wellbeing [31,48]. Second, while the EBIC-graphical LASSO regularization procedure employed here is appropriate for exploratory network estimation, it may set small true edges to zero and is sensitive to the regularization hyperparameter [49]. Third, the spirituality constructs were operationalized as two dimensions of a single instrument, and future work using more comprehensive measures may reveal additional differentiation within the spiritual cluster. Notwithstanding these limitations, the present findings advance understanding of the interrelations between yoga, health, and psychological functioning by highlighting WHO-5 wellbeing, DASS-21 depression, and SHALOM Experiential as prominent nodes within this network, albeit on different centrality metrics.

The hierarchical regression complemented the network analysis by identifying variables that retained unique associations with subjective wellbeing after accounting for shared variance among predictors. Whereas the network analysis characterized the conditional association structure among yoga practice, perceived health, wellbeing, distress, and spirituality, the regression analysis identified variables independently associated with wellbeing. Together, these complementary approaches provide both a system-level perspective and an outcome-specific perspective on wellbeing [46,70].

A key finding shared by both analyses was the importance of distress-related variables. The network revealed a tightly connected distress cluster, particularly between anxiety and stress, while the regression analysis showed that depressive symptoms and stress remained uniquely associated with wellbeing after accounting for spirituality, perceived health, and yoga practice frequency. These findings are consistent with previous research indicating that depression, anxiety, and stress represent related but distinct dimensions of psychological distress associated with subjective wellbeing [41,46,60]. Anxiety did not retain a unique association with wellbeing in the regression model, despite its strong network connectivity, likely reflecting shared variance with stress. The acceptable VIF values support this interpretation by indicating that multicollinearity was not sufficiently high to compromise coefficient estimation.

The regression analysis further demonstrated that perceived health and yoga practice frequency retained unique associations with wellbeing beyond distress-related variables. Together with the network findings, this suggests that wellbeing among yoga practitioners is related not only to lower psychological distress but also to more positive perceptions of health and more frequent yoga practice. More specifically, the present findings are consistent with WHO frameworks that conceptualize wellbeing as multidimensional [5]. The results suggest that yoga-based health promotion may warrant further examination of both distress reduction and the cultivation of positive health perceptions, regular practice, and environmental spirituality. Given yoga’s growing recognition as a health-related behavior within physical activity promotion [12,56], these findings support the integration of yoga into community wellbeing initiatives, though cross-cultural replication is warranted.

In the present study, spirituality was operationalized using the SHALOM framework [71], which conceptualizes spiritual wellbeing as a multidimensional construct encompassing personal, communal, environmental, and transcendental domains. Environmental spirituality refers to a sense of harmony and connectedness with nature and the broader environment, whereas transcendental spirituality reflects one’s relationship with a higher power, divine reality/God, or a transcendent dimension. The findings related to spirituality are another area where the regression adds nuance. In the network, spirituality did not function uniformly: some spiritual dimensions were positively integrated into the broader system, while others showed more complex connections. The regression clarifies this by indicating that the positive association with wellbeing was specific to the experiential environmental domain rather than the experiential transcendent domain, whereas in the network it was the global experiential score (not the global ideal score) that was more central. Together, these results underscore the relevance of lived, experiential spirituality—and its environmental dimension in particular—over idealized spiritual standards.

The differential pattern observed in the main model—where environmental spirituality emerged as a significant positive predictor of wellbeing, while transcendent spirituality did not—may reflect differences in how these dimensions relate to everyday psychological functioning. Because this environmental-specific association was not robust when all four experiential domains were modeled together (Table S8), the interpretation that follows is offered tentatively. This pattern is consistent with a growing body of research on nature connectedness, which demonstrates that individuals who experience a sense of unity with the natural environment tend to report higher levels of wellbeing, likely due to enhanced positive affect, meaning, and restorative processes [72,73,74]. Further recent evidence suggests that engagement with natural environments is associated with improved mental health and emotional regulation, particularly through pathways involving stress reduction and attentional restoration [75,76]. This may be especially relevant in the context of yoga practice, which often emphasizes embodied awareness and mindful engagement with the surrounding environment, thereby potentially strengthening the link between environmental connectedness and wellbeing [21,77]. A plausible explanatory pathway underlying these associations may involve stress reduction, attentional restoration, and enhanced emotional regulation, whereby engagement with natural environments and embodied practices such as yoga facilitates recovery from cognitive fatigue and promotes positive affect, which in turn may be positively associated with wellbeing [21,76,78].

In contrast, more abstract or transcendent spiritual orientations may operate through context-dependent or belief-based pathways that are less directly reflected in measures of everyday affective wellbeing, such as the WHO-5. Environmental spirituality, which emphasizes lived experience and connection to nature, may therefore show more proximal associations with wellbeing, whereas transcendent spirituality may be more distal, culturally mediated, or variably interpreted across individuals. This distinction aligns with literature indicating that spirituality can support mental health through meaning, connectedness, and coping, while also suggesting that experiential and relational aspects may have more immediate implications for wellbeing than belief-oriented or transcendent dimensions [55,65]. More recent research further supports this differentiation, highlighting that spirituality–wellbeing associations are not uniform across dimensions, with some forms—particularly those grounded in lived experience and contextual meaning-making—showing more consistent links to mental health outcomes, while others may be weaker or contingent on individual and sociocultural factors [24,79].

Overall, the regression strengthens the paper by providing a clearer account of what the network alone cannot show. The network identifies the architecture of associations, central nodes, and conditional clustering, but it does not indicate which variables are the strongest unique correlates of wellbeing when all predictors are modeled together. The regression addresses that gap. Taken together, the two analyses suggest that wellbeing in yoga practitioners is best understood as a multidimensional outcome uniquely associated with distress, perceived health, yoga engagement, and spirituality, with the regression helping specify which of these factors have direct associations with the outcome variable [46,55,56].

It is also important to note that the sample was predominantly female, reflecting typical participation patterns in yoga research but potentially influencing the observed associations, particularly those related to wellbeing and spirituality. Gender differences in these domains have been reported in the previous literature; therefore, the present findings should be interpreted with caution when generalizing to more gender-balanced populations.

### Limitations

Several limitations should be acknowledged. First, the cross-sectional design precludes causal inference. Therefore, network edges should be interpreted as conditional associations, and regression “predictors” as statistical rather than causal predictors. Longitudinal or experimental studies are needed to clarify directionality. Second, convenience and snowball sampling may limit generalizability, and recruitment through social networks and yoga organizations may have introduced self-selection bias. Furthermore, recruitment through yoga associations and related social networks may have resulted in the overrepresentation of individuals who are more actively engaged in yoga practice and spirituality. Consequently, the observed pattern of associations may not fully reflect the broader population of yoga practitioners with more diverse backgrounds, motivations, or levels of engagement. Third, all variables were self-reported, making the findings vulnerable to recall bias, social desirability, and shared method variance; moreover, perceived health was assessed with a single item, which may not fully capture health status. Moreover, most participants (82.5%) reported engaging in physical activity in addition to yoga; consequently, the observed associations between perceived health and wellbeing cannot be attributed specifically to yoga practice, because other forms of physical activity may also have contributed to them. Fourth, the sample consisted mainly of highly educated female participants, which may have influenced the observed associations, particularly those involving spirituality and wellbeing, as previous research suggests gender differences in these domains [37]. Fifth, the study lacked a comparison group; thus, no conclusions can be drawn about the exclusivity of the results to yoga practitioners. Sixth, nearly one-third of participants were yoga instructors, who may differ from non-instructor practitioners in practice intensity, identity, spirituality, and wellbeing. This potential difference was not examined in the present study and warrants further investigation. Finally, although recruitment was nationwide, the study was conducted in Hungary, limiting cross-cultural generalizability and warranting replication in more diverse samples. Furthermore, because data collection extended over more than two years, temporal variation in participant characteristics cannot be entirely excluded. However, the same recruitment procedures and eligibility criteria were applied throughout the study period.

There were also several limitations in the analytical approach. First, yoga practice was measured primarily by frequency, with no detailed assessment of intensity, duration, style, or practice context, limiting conclusions about qualitative differences in yoga engagement. Second, spirituality was operationalized differently across analyses: the network model used global SHALOM-derived ideal and experiential spirituality scores, whereas the regression included selected domain-specific dimensions, namely environmental and transcendent spiritual health (an exploratory, theoretically motivated selection). This difference may limit direct comparability between the two analytical approaches and may have obscured other domain-specific patterns. Indeed, a sensitivity analysis entering all four experiential domains simultaneously (Table S8) indicated that the experiential personal domain, rather than the environmental domain, was the unique positive correlate of wellbeing, suggesting that the environmental-specific association is not robust to the inclusion of the personal domain and should be interpreted with caution. The broader positive association between experiential spirituality and wellbeing was nonetheless confirmed by the global experiential score. Third, the network-node selection was based on theoretical relevance; thus, the exclusion of other plausible variables such as sleep, social support, or practice motivation may have limited the representation of the network. Fourth, although EBIC-graphical LASSO is suitable for sparse networks, it can shrink small meaningful edges to zero and is sensitive to model specification. Additionally, betweenness centrality showed only moderate stability. These limitations call for further research with more balanced samples and, ideally, longitudinal designs.

## 5. Conclusions

This study contributes to a better understanding of wellbeing, psychological distress, perceived health, and spirituality among yoga practitioners by highlighting their complex pattern of conditional associations. Subjective wellbeing emerged as a central node within the estimated network and was most consistently associated with better perceived health and lower psychological distress, particularly lower depression and stress. Yoga practice frequency showed more indirect associations with wellbeing, primarily through its connections with perceived health and subjective wellbeing. The findings also suggest that spirituality is not uniformly protective: the experiential environmental domain was positively associated with wellbeing in the regression, whereas the experiential transcendent domain was not, and global ideal spirituality showed negative or more complex associations in the network. The positive environmental association was, however, evident only in the main model and was not robust to the inclusion of the other experiential domains, so it should be regarded as preliminary. From a public health perspective, these findings highlight the potential relevance of considering regular yoga practice, perceived health, psychological distress, and meaningful spiritual experiences together when investigating wellbeing among yoga practitioners. Because the study was cross-sectional, the findings should be interpreted as associations rather than causal effects, and longitudinal and cross-cultural research is needed to confirm the temporal stability, directionality, and generalizability of these relationships.

## Figures and Tables

**Table 1:** Sociodemographic and yoga-related characteristics of the participants (n = 326).

Age	Mean + SD (Min–Max) 39.84 ± 14.81 (18–82)	
	n	%
Gender		
Male	19	5.8
Female	307	94.2
Educational level		
Elementary school	1	0.3
Vocational school	2	0.6
Graduation (high- or vocational school)	106	32.5
BSc or MSc degree	208	63.8
PhD or higher degree	9	2.8
Family status		
Single	81	24.8
In a relationship or civil partnership	80	24.5
Married	134	41.1
Divorced	24	7.4
Widowed	7	2.1
Place of residence		
Village	42	12.9
Small town	25	7.7
City (10,000–100,000 inhabitants)	87	26.7
Large city (>100,000 inhabitants)	48	14.7
Capital city	124	38.0
Country		
Hungary	312	95.7
Other countries	14	4.3
Region (Hungary)		
Pest county (incl. capital region)	178	54.6
Other Hungarian counties	134	41.1
Foreign countries	14	4.3
Employment status		
Student	65	19.9
Entrepreneur	74	22.7
Employed (employee/public servant)	151	46.3
Casual worker	4	1.2
Retired/unemployed/other	32	9.8
Yoga-related characteristics		
Yoga practitioner	224	68.7
Yoga instructor	102	31.3
Additional physical activity (besides yoga)		
No	57	17.5
Yes	269	82.5

Note. Values are presented as frequencies (n) and percentages (%), unless otherwise indicated. SD = standard deviation. The sample contains no missing data.

**Table 2:** Standardized Centrality Indices per Node (Sorted by Betweenness).

Node	Betweenness	Closeness	Strength	Expected Influence
WHO-5 Wellbeing	1.874	1.958	0.884	−1.395
DASS-21 Depression	1.142	0.752	0.798	−0.227
SHALOM Experiential	0.228	0.098	0.983	1.805
SHALOM Ideal	−0.503	−0.349	0.269	0.595
DASS-21 Stress	−0.685	−0.313	0.215	0.311
DASS-21 Anxiety	−0.685	−0.494	−0.333	0.400
Perceived Health	−0.685	−0.209	−0.947	−0.641
Yoga Frequency	−0.685	−1.444	−1.868	−0.849

Note. All values are standardized z-scores computed from the estimated network. Betweenness = proportion of shortest paths in the network that pass through a node; Closeness = inverse of the mean shortest path length to all other nodes; Strength = sum of absolute edge weights; Expected Influence = sum of signed edge weights (sensitive to edge direction).

**Table 3:** Final Hierarchical Regression Model Predicting WHO-5 Wellbeing.

Predictor	B	SE B	β	t	*p*	95% CI for B	VIF
SHALOM_B Environmental	0.080	0.028	0.136	2.85	0.005	[0.025, 0.135]	1.39
SHALOM_B Transcendent	0.017	0.018	0.043	0.93	0.354	[−0.019, 0.053]	1.29
Depression	−0.228	0.042	−0.362	−5.41	<0.001	[−0.310, −0.145]	2.74
Anxiety	0.072	0.043	0.104	1.68	0.094	[−0.012, 0.156]	2.37
Stress	−0.116	0.039	−0.198	−2.98	0.003	[−0.192, −0.039]	2.69
Weekly yoga frequency	0.145	0.059	0.104	20.44	0.015	[0.028, 0.261]	1.12
Perceived health	1.087	0.186	0.256	5.86	<0.001	[0.721, 1.452]	1.17

Note. Dependent variable = WHO-5 wellbeing; SHALOM_B = SHALOM Experiential level; β = standardized coefficient; SE = standard error.

## Data Availability

The data used in this study are deposited at Mendeley Data (DOI: https://doi.org/10.17632/sb8b8mgcv7.1). A retrospective registration of the study design and analytical approach was completed on the Open Science Framework (OSF) prior to manuscript submission. Because this registration was retrospective rather than prospective, it improves the transparency of the reported design and analyses but does not carry the same evidentiary weight as prospective preregistration. The registration is available at https://doi.org/10.17605/OSF.IO/JRYVU.

## Supplementary Materials

The following supporting information can be downloaded at https://www.mdpi.com/article/10.3390/healthcare14152293/s1, Table S1. Frequency of participation in yoga components (n = 326). Table S2. Summary of Network Structure. Table S3. Edge Weight Matrix: Regularized Partial Correlations. Table S4. Standardized Clustering Coefficients per Node. Table S5. Bootstrap Procedure Summary. Table S6. Correlation-Stability (CS) Coefficients for Centrality Indices. Table S7. Hierarchical Regression Model Summary Predicting WHO-5 Wellbeing. Table S8. Sensitivity Analyses for the Hierarchical Regression Predicting WHO-5 Wellbeing: Alternative Operationalizations of Experiential Spirituality. Figure S1 Bootstrapped Edge-Weight Confidence Intervals (500 Nonparametric Bootstraps). Note. Each horizontal row shows the bootstrapped 95% confidence interval (gray band) and bootstrap mean (black line/dot) for one edge, plotted against the original sample estimate (red line/dot). Edges are ordered from strongest (top) to weakest (bottom) by absolute weight. Narrower bands indicate more precisely estimated edges.

## Abbreviations

The following abbreviations are used in this manuscript:

CS	Correlation stability
DASS-21	Depression Anxiety Stress Scales
EBIC	Bayesian information criterion
GGM	Gaussian graphical model
SHALOM	Spiritual Health and Life-Orientation Measure
SWB	Subjective wellbeing
VIF	Variance inflation factors
WHO	World Health Organization
WHO-5	WHO Well-Being Index
