# Supplementary material for: Mapping Conditional Associations Among Wellbeing, Distress, Spirituality, and Yoga Practice: A Network Analysis
Source: Healthcare (Basel). 2026 Jul 29;14(15):2293. doi: 10.3390/healthcare14152293 (PMC13464920; doi:10.3390/healthcare14152293)
Supplement: Supplementary file 1 [file healthcare-14-02293-s001.zip › healthcare-4414238-supplementary.pdf]

Supplementary Materials

Mapping Wellbeing, Distress, and Spirituality in Yoga Practitioners: A Network Analysis

Table S1. Frequency of participation in yoga components (n = 326).

Frequency	Asana (n, %)	Pranayama (n, %)	Relaxation (n, %)	Meditation (n, %)
Once a week	103 (31.6%)	126 (38.7%)	121 (37.1%)	179 (54.9%)
2 times a week	65 (19.9%)	66 (20.2%)	64 (19.6%)	38 (11.7%)
3 times a week	62 (19.0%)	46 (14.1%)	55 (16.9%)	34 (10.4%)
4 times a week	28 (8.6%)	22 (6.7%)	25 (7.7%)	21 (6.4%)
5 times a week	24 (7.4%)	22 (6.7%)	15 (4.6%)	15 (4.6%)
6 times a week	11 (3.4%)	10 (3.1%)	6 (1.8%)	7 (2.1%)
Daily	33 (10.1%)	34 (10.4%)	40 (12.3%)	32 (9.8%)
Total	326 (100%)	326 (100%)	326 (100%)	326 (100%)

Note. Asana = yoga poses, Pranayama = breathing exercises. The sample contains no missing data.

Table S2. Summary of Network Structure.

Nodes	Non-zero edges	Sparsity
8	20 of 28 possible	0.286

Table S3. Edge Weight Matrix: Regularized Partial Correlations.

Node	Yoga	P.Health	WHO-5	Dep.	Anx.	Stress	Sp.Id.	Sp.Exp.
Yoga Frequency	—							
Perceived Health	0.348	—						
WHO-5 Wellbeing	0.349	0.412	—					
DASS-21 Depression	-0.087	0.000	0.000	—				
DASS-21 Anxiety	0.000	-0.094	-0.004	0.105	—			
DASS-21 Stress	0.000	0.015	0.000	-0.041	0.686	—		
Spiritual (Ideal)	0.000	-0.222	-0.142	0.288	0.237	-0.104	—	
Spiritual (Experiential)	0.000	0.000	-0.027	0.095	0.018	0.101	0.122	—

Note. Values are regularized partial correlations between each node pair as estimated by the EBIC-graphical LASSO [49]. Zeros indicate edges regularized to zero (absent from the network). Dashes (—) on the diagonal represent undefined self-associations. Dep. = DASS-21 Depression; Anx. = DASS-21 Anxiety; P.Health = Perceived Health; Sp.Id. = Spiritual (Ideal); Sp.Exp. = Spiritual (Experiential).

Table S4. Standardized Clustering Coefficients per Node.

Node	Barrat	Onnela	WS	Zhang
DASS-21 Anxiety	-1.492	1.792	-2.052	1.825
DASS-21 Depression	-0.637	0.533	-0.324	0.125
DASS-21 Stress	-0.823	-0.570	-0.324	1.104
Perceived Health	-0.294	-0.959	-0.324	-0.650
Spiritual (Experiential)	0.677	-0.461	0.540	-0.983
Spiritual (Ideal)	1.566	0.381	0.972	-0.477
WHO-5 Wellbeing	0.194	0.549	0.540	-0.905
Yoga Frequency	0.809	-1.264	0.972	-0.038

Note. All values are standardized z-scores. Four clustering estimators are reported: Barthélemy et al. [51], Onnela et al. [52], Watts–Strogatz [53], and Zhang & Horvath [54]. Discrepancies across estimators reflect differences in how each algorithm weights edge magnitudes.

Table S5. Bootstrap Procedure Summary.

Bootstrap type	N bootstraps	Purpose
Nonparametric	500	Edge accuracy
Case-dropping	500	Centrality stability (CS-coefficient)

Note. Two separate bootstrap procedures were conducted to assess edge accuracy and centrality stability, respectively.

Table S6. Correlation-Stability (CS) Coefficients for Centrality Indices.

Centrality index	CS-coefficient	Interpretation
Strength	0.70	Excellent (≥ 0.50 threshold met)
Closeness	0.60	Excellent (≥ 0.50 threshold met)
Betweenness	0.40	Acceptable (≥ 0.25 threshold met)

Note. The CS coefficient is the maximum proportion of cases that can be removed while, with 95% probability, retaining a correlation of at least .70 between centrality estimates from the original sample and those from subset samples [50].

Table S7. Hierarchical Regression Model Summary Predicting WHO-5 Wellbeing.

Step	Predictors entered	R^2	Adj. R^2	ΔR^2	ΔF	df	p
1	SHB Environmental	.159	.157	.159	61.34	1, 324	< .001
2	+ SHB Transcendent	.169	.164	.010	3.97	1, 323	.047
3	+ Depression, Anxiety, Stress	.407	.398	.238	42.83	3, 320	< .001
4	+ Weekly yoga frequency	.425	.414	.017	9.59	1, 319	.002
5	+ Perceived health	.481	.469	.056	34.28	1, 318	< .001

Note. Final model: $F(7, 318) = 42.04$, $p < .001$; Durbin-Watson = 2.10.

Table S8. Sensitivity Analyses for the Hierarchical Regression Predicting WHO-5 Wellbeing: Alternative Operationalizations of Experiential Spirituality.

**Predictor**	**B**	**SE B**	**β**	**t**	**p**	**95% CI**	**VIF**
**Model A. All four experiential SHALOM domains entered simultaneously**							
SHALOM_B Personal	0.124	0.043	.195	2.89	.004	[0.040, 0.209]	2.88
SHALOM_B Communal	0.027	0.039	.037	0.69	.488	[−0.050, 0.105]	1.82
SHALOM_B Environmental	0.001	0.035	.002	0.03	.974	[−0.068, 0.070]	2.27
SHALOM_B Transcendent	0.011	0.018	.028	0.63	.531	[−0.024, 0.047]	1.30
Depression	−0.189	0.043	−.301	−4.41	< .001	[−0.274, −0.105]	2.95
Anxiety	0.074	0.042	.108	1.76	.079	[−0.009, 0.157]	2.37
Stress	−0.132	0.039	−.224	−3.37	< .001	[−0.208, −0.055]	2.81
Weekly yoga frequency	0.143	0.058	.104	2.46	.015	[0.029, 0.258]	1.13
Perceived health	0.990	0.185	.233	5.36	< .001	[0.627, 1.354]	1.20
**Model B. Global experiential spirituality score**							
Global experiential spirituality	0.036	0.008	.200	4.42	< .001	[0.020, 0.052]	1.28
Depression	−0.209	0.042	−.332	−4.96	< .001	[−0.292, −0.126]	2.81
Anxiety	0.069	0.042	.101	1.65	.099	[−0.013, 0.152]	2.35
Stress	−0.124	0.038	−.211	−3.25	.001	[−0.199, −0.049]	2.65
Weekly yoga frequency	0.131	0.058	.094	2.26	.025	[0.017, 0.245]	1.10
Perceived health	1.024	0.185	.241	5.54	< .001	[0.661, 1.388]	1.18

Note. Dependent variable = WHO-5 wellbeing (N = 326). Both models are exploratory sensitivity analyses that replace the two experiential SHALOM domains used in the main model (Table 4) with (Model A) all four experiential domains entered simultaneously and (Model B) the single global experiential spirituality score, while retaining the same depression, anxiety, stress, weekly yoga frequency, and perceived-health predictors. Model A: $R^2 = .501$, adjusted $R^2 = .487$, $F(9, 316) = 35.28$, $p < .001$. Model B: $R^2 = .491$, adjusted $R^2 = .482$, $F(6, 319) = 51.33$, $p < .001$. At the bivariate level, all four experiential domains were positively and significantly correlated with wellbeing (personal $r = .50$, environmental $r = .40$, communal $r = .36$, transcendent $r = .26$; all $p < .001$), as was the global experiential score ($r = .47$, $p < .001$). When all four domains were entered together (Model A), the experiential personal domain emerged as the unique positive predictor, whereas the environmental domain was attenuated to non-significance, reflecting the substantial shared variance between the personal and environmental domains ($r = .72$). The negative associations of depression and stress and the positive associations of weekly yoga frequency and perceived health remained stable across all models. SHALOM_B = experiential (lived) level of the SHALOM; β = standardized coefficient; SE = standard error; CI = confidence interval; VIF = variance inflation factor.

Supplementary Figure

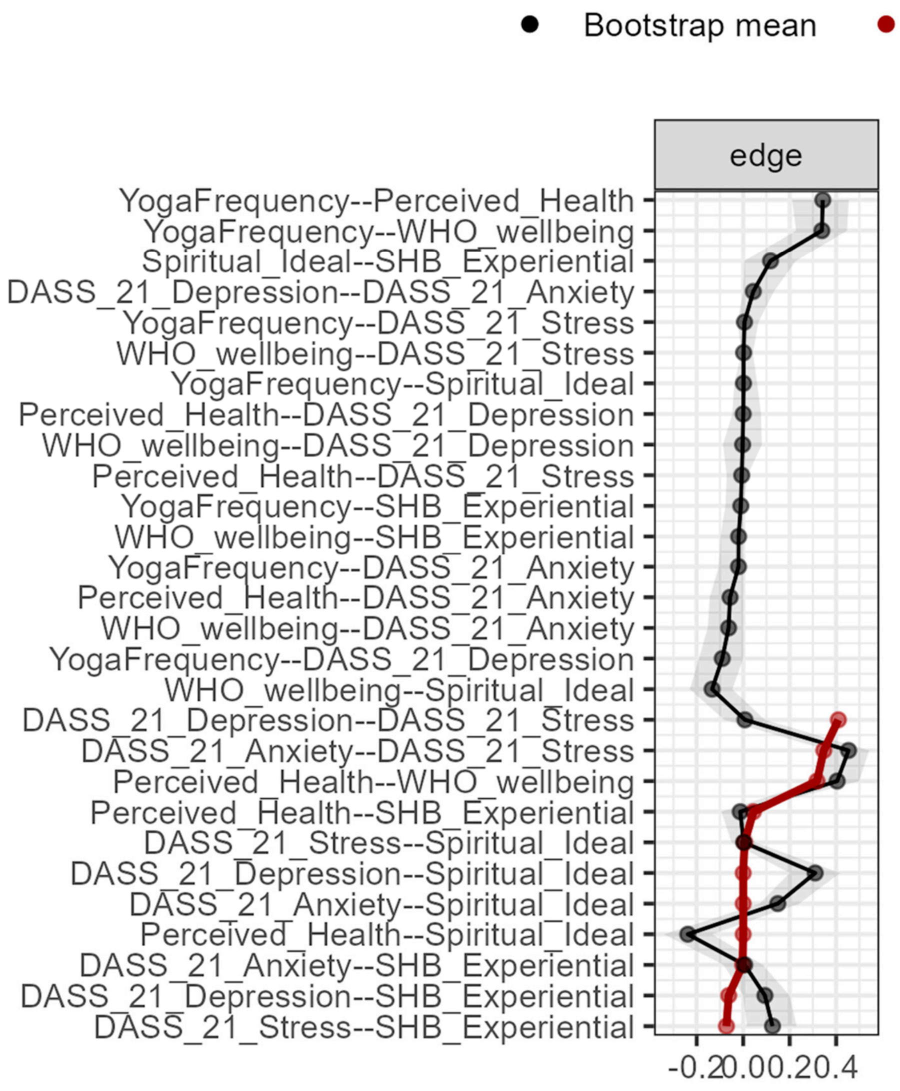

Figure S1 Bootstrapped Edge-Weight Confidence Intervals (500 Nonparametric Bootstraps).

Note. Each horizontal row shows the bootstrapped 95% confidence interval (gray band) and bootstrap mean (black line/dot) for one edge, plotted against the original sample estimate (red line/dot). Edges are ordered from strongest (top) to weakest (bottom) by absolute weight. Narrower bands indicate more precisely estimated edges.
